# Comment on the paper "Cost-effectiveness of sofosbuvir in hepatitis C genotype 1 infection in Germany: A reanalysis of published results"

**DOI:** 10.1371/journal.pone.0245480

**Published:** 2021-02-18

**Authors:** Axel C. Mühlbacher, Andrew Sadler

**Affiliations:** 1 Health Economics and Health Care Management, Hochschule Neubrandenburg, Neubrandenburg, Germany; 2 Gesellschaft für Empirische Beratung GmbH (GEB), Freiburg, Germany; Public Library of Science, UNITED KINGDOM

## Introduction

The author of the paper to be discussed claims to have reanalyzed a case study on the efficiency frontier (EF). Gandjour discusses the rationale of the EF and presumes a potential publication bias [1]. We as the authors of the respective paper welcome the methodological discussion beyond the peer-review process, and we generally acknowledge the critical appraisal of published articles as a scientific imperative. Against this background we acknowledge that Afschin Gandjour in particular has rendered outstanding services in dedicating his career to publishing commentaries and letters on original research.

Despite these unparalleled merits, we criticize the misinterpretation, the execution and are puzzled about the limited coverage of the methodological approach. It is in the nature of the publication bias that we do not want to argue against the accusation that the outcome of our research study influenced the decision to publish. We cannot prove the opposite.

## Value assessment framework and efficiency frontier

However, we wish to stress that our case study was published to demonstrate the application of patient preference information (PPI) in the efficiency frontier (EF). We have repeatedly pointed out that our goal was to demonstrate the operationalization of a composite indicator (single-dimensional measure of overall benefit) considering uncertainty and combining clinical data and preference data in a value assessment framework (VAF). We clearly state, that we aim to demonstrate how an EF can be used to inform decision makers about “whether a treatment is efficient at given prices demonstrated through a case study on chronic hepatitis C“. For this purpose, we published a case study for the practical implementation of the efficiency frontier to address methodological issues of scoring, weighting and aggregation.

## Uncertainty analysis with different value functions

The author cast doubts about the scientific integrity of the papers, by stating that “it is not possible to retrieve information about potential differences from their texts” and that there were only “small differences […] that appear only to be the result of different sets of draws in the Monte-Carlo simulation” [1]. In fact, there are several rather significant differences in the methodological approaches which are described and presented in detail in the papers [2, 3]. Already at this point we must express our astonishment that these differences were not recognized while studying the underlying literature. A thorough reading or even just comparing the number of endpoints used in the two models (see Table 3 in both papers) should have revealed the significant differences in the study design and data. Gandjour did not report that the models differed in their scope, in the number of clinical endpoints and they were based on different distributional assumptions and different preference data. The German paper [3] describes a reduced model with older preference data from the IQWiG pilot study [4, 5] to identify, weight, and prioritize multiple attributes in the indication hepatitis C, while the English paper [2] describes a more comprehensive model with data from a more recent patient preference study [6].

## Addressed limitations of the efficiency frontier analysis

The points addressed in this comment were to a large extent part of our own limitation section. We agree that different clinical data might shift the efficiency frontier and lead to a different result, as might the consideration of other treatment alternatives. We would have welcomed a more thorough analysis of the current clinical evidence. Without guaranteeing completeness or drawing conclusions we have carried out a recent literature review, finding 39 papers (please see S1 Fig in S1 Appendix). We stated that our results cannot be used without restriction. For example, patient preference data was obtained from patients with experiences and attitudes difficult to compare from today’s perspective. We also mentioned in the limitation section that our study does not address how the aggregation of different patient groups is done. We explicitly address the risk that significant patient groups are not included in the analysis and that an overall assessment cannot be derived. In order to illustrate the methodological approach, we focused on GT1 TN without cirrhosis. We also stated that we applied a simplified aggregation rule and that more complex functions should be tested in future evaluations. We also addressed a possible heterogeneity regarding patient preferences in the study sample that was not considered in our analysis and indicated that the consideration of long-term costs and outcomes could lead to a different result [2, 3].

We agree that it is important to consider the time component when creating or interpreting the efficiency frontier. We believe that the efficiency frontier has been drawn correctly. In our analysis Gilead products did not differentiate significantly in effectiveness from Viekirax/Exviera [2, 3]. The most efficient compound resulting from the efficiency frontier was used as an example to calculate a net monetary benefit (NMB) and a price acceptability curve (PAC). In this case this was Harvoni 8w.

## The CHEERS checklist

Eventually the 24-item CHEERS checklist was used to appraise the completeness of reporting of the case study [7]. The paper only mentions that there was one item on the list that was not fully met in our study (Item No 11 “Measurement of effectiveness”) [1]. Although the percentage of ticks on a checklist is not in itself a good measure whether the methodological quality of a study is good or bad [8], but not meeting only one criterion on the checklist seems like a good percentage. However, at the end of the paper it was not discussed whether other clinical data would be more appropriate and lead to a different result regarding the efficiency frontier. But even this evaluation would have been possible, admittedly with some effort. After all, in our papers we have described the data used and how they were used in detail. With some effort, a carryover with our data would have been possible and the data could have been compared with the clinical evidence now available. (see S1 Appendix).

The weighted clinical data could have been varied in every respect to document a possible effect on the outcome and to see how results would flip. Anyway, we acknowledge that our reporting on the methods used for identification of included studies was incomplete (due to the limited number of studies available at the time of publication). But we disagree with the appraisal regarding item No 12 “Measurement and valuation of preference-based outcomes described” and item No 18 “Study parameters described” which was reported as not meet (N) in Table 5 of the comment [1]. We are particularly surprised by the appraisal on item No 18 as we provided a downloadable appendix accompanying our paper which comprehensively describes the study parameters and references.

## Patient preference information (PPI) and health technology assessment (HTA)

We hoped to contribute to the methodological discussion of how to implement patient preference information (PPI) in Health Technology Assessment (HTA). We aimed at the aggregation of clinical and preference data to establish a composite measure of overall benefit in a transparent and comprehensible VAF. Calculations were transparent and clinical and preference data were accessible. We welcome any critical and constructive discussion or enhancement of our published model. In this case the quality of this contribution depends not only on what is being addressed, rather on what is not being discussed or addressed.

In accordance to the Multi-Criteria Decision Analysis (MCDA) literature we applied and described the following steps in our VAF [9]:

1. definition of the decision problem to identify alternatives and decision tasks;
2. identification of relevant indicators and specification of the decision model;
3. performance measurement of each indicator;
4. scoring of indicators (normalization);
5. weighting of normalized indicators;
6. aggregation of indicators;
7. interpretation and analysis of uncertainty.

With the recombination of already published cost and effectiveness data only the last step is addressed in the paper. We had hoped for a more ambitious criticism, as each of these steps 1–6 requires a careful and cautious approach and has its limitations. Modifications in all remaining steps, especially scoring, weighting and aggregation will significantly affect the results. Against the background of the insinuations made, we would have expected more methodological depth and consider the uncritical acceptance of all our calculations to be questionable. Gandjour’s paper might have added some information when the models would have been adjusted to the current status of literature and the prices. In our opinion, a recent publication should have been reflective of the current developments.

## Keeping up the critical discussion

After reading this paper, we are more convinced than ever that we need a discussion on a generic framework for calculating a multi-dimensional overall composite value index to support health technology assessment (HTA) assisting decision modelers and decision experts. Researchers should be able to easily adapt the framework to add relevant elements that are specific and, such as disease-specific clinical or preference data. Transparent VAF would therefore help to critically reflect and add new information. Our papers were intended to introduce and push forward this methodological discussion on aggregation, weighting, normalization of effects, consideration and interpretation of uncertainty and the use of preference data in HTA models.

We hope and encourage a more detailed discussion about the implementation and use of value assessment frameworks in the future.

## Supporting information

S1 Appendix (DOCX)
