# Supplementary material for: Comment on the paper "Cost-effectiveness of sofosbuvir in hepatitis C genotype 1 infection in Germany: A reanalysis of published results"
Source: PLoS One. 2021 Feb 18;16(2):e0245480. doi: 10.1371/journal.pone.0245480 (PMC7891755; doi:10.1371/journal.pone.0245480)
Supplement: S1 Appendix — (DOCX) [file pone.0245480.s001.docx]

#### **S1 Appendix**

Until 2011, treatment regimens based on pegylated interferon alpha (PEG-IFN-α) in combination with ribavirin (RBV) were the standard therapy for hepatitis C [1], but these treatment regimens have become a thing of the past due to their severe side effect profile (including headache, fatigue, flu-like illness and hematological toxicity) and comparatively low cure rates [2]. In 2011, first generation protease inhibitors telaprevir (TVR) and boceprevir (BOC), and later simeprevir and the polymerase inhibitor sofosbuvir, were approved and used in combination with pegylated interferon alpha (PEG-IFN-α) and ribavirin (RBV) [3]. Since 2014, interferon-free therapies have also been approved for the treatment of hepatitis C, which are supposed to reduce the duration of treatment and side effects as well as yielding higher sustained virologic response rates (SVR) and quality-adjusted life years (QALYs), that the long-term health economic outcomes of SOF-based programs (Sovaldi®) and an alloral pegIFN-free and RBV-free LDV/SOF regimen (Harvoni®) are cost-effective and lead to favorable short and long-term clinical and health economic outcomes compared to current therapies or the old standard of care (SOC) [3-5] . In addition, some studies show that newer treatment regimens, such as Elbasvir/ Grazoprevir (EBR/GZR) or ombitasvir/paritaprevir/ritonavir and dasabuvir (Viekira Pak®) with or without ribavirin are also cost-effective and, in some cases, dominate previous therapies such as Harvoni® (SOF/LDV) or Daklinza® + Sunvepra® (DCV/ASV) [6-8]. However, Sofosbuvir/ Ledipasvir (Harvoni®) is not more cost-effective compared to Ombitasvir/ Paritaprevir/Ritonavir and dasabuvir (Viekira Pak®) due to the higher price (except possibly in genotype 1 patients with cirrhosis), Harvoni® could become more cost effective by lowering the price [8]. See table 1 for an overview of cost-effectiveness studies and S1 Fig for the associated database search.

|  | **With interferon** | | | | |
| --- | --- | --- | --- | --- | --- |
| **Active agent**  **Manufacturer/ trade name**  **Abbreviation** | **pegylated interferon-alfa and ribavirin**  **PEGasys®+ Copegus®**  **PR** | **pegylated interferon-alfa and ribavirin+ boceprevir**  **PEGasys®+ Copegus®+**  **Victrelis®**  **PR+ BOC** | **pegylated interferon-alfa and ribavirin + telaprevir**  **PEGasys®+ Copegus®+**  **Incivo^®^**  **PR+TEL** | **pegylated interferon-alfa and ribavirin+ sofosbuvir**  **PEGasys®+ Copegus®+**  **Sovaldi®**  **SOF+PR** | **pegylated interferon-alfa and ribavirin+ simeprevir**  **PEGasys®+ Copegus®+ Olysio®**  **SMV+PR** |
| **Cost-effective compared to** |  | **PR**  [9] [10] [1] [11] [12] [13] [14] [15] [16] | **PR**  [9] [1] [11] [12] [13] [14] [2] [17]  **PR+ BOC**  [11] [14] | **PR**  [3, 12, 18]  **PR+ BOC** [18] [19] [3]  **PR+TEL** [18] [19]  **SMV+PR** [18] | **PR** [20]  **PR+TEL**  [20] [21] |
| **Not Cost-effective compared to** | **PR+BOC**  [9] [10] [1] [11] [12] [13] [14] [15] [16]  **PR+TEL** [9] [1] [11] [12] [13] [14] [2] [17]  **SOF+PR**  [3, 12, 18]  **SOF+RBV** [22] [23] [24] [25] [26]  **SOF+SMV** [23]  **DCV/ASV** [27] [28] [29]  **3D+RBV** [30] [8]  **EBR/GZR** [31]  **SMV+PR** [20]  **SOF+DCV** [32] | **SOF+PR** [18] [3] [19]  **PR+TEL**  [11] [14]  **SOF/ LDV** [33] [5]  **SOF+RBV** [22] [23]  **SOF+SMV** [23]  **DCV/ASV** [27] [29] | **SOF+PR** [18] [19]  **SOF/ LDV** [21] [8] [33] [34]  **SOF+RBV** [22] [25]  **DCV/ASV** [27] [29]  **SMV+PR** [20] [21]  **3D+RBV** [8]  **Harvoni**  [8] | **SOF/ LDV**  [5] [4] | **SOF+PR** [18] |

|  | **Without interferon** | | | | | | | | | |
| --- | --- | --- | --- | --- | --- | --- | --- | --- | --- | --- |
| **Active agent**  **Manufacturer/ trade name**  **Abbreviation** | **Sofosbuvir+ ribavirin *^1^**  **Sovaldi®+ Copegus®**  **SOF+RBV** | **Sofosbuvir+ daclatasvir**  **Sovaldi®+**  **Daklinza®**  **SOF+DCV**  **(*+RBV)** | **sofosbuvir/ simeprevir*^1^**  **Sovaldi®+ Olysio® (± Copegus®)**  **SOF+SMV (± RBV)** | **Sofosbuvir/ ledipasvir**  **(± ribavirin)**  **Harvoni®**  **SOF/ LDV (± RBV)** | **sofosbuvir/ velpatasvir**  **Sovaldi®+**  **Epclusa®**  **SOF/VEL** | **Daclatasvir/ Asunaprevir**  **Daklinza® +**  **Sunvepra®**  **DCV/ASV** | **ombitasvir, paritaprevir, ritonavir**  **Exviera^®^**  **OBV/PTV/r** | **ombitasvir, paritaprevir, ritonavir and dasabuvir**  **Viekira Pak®**  **3D** | **ombitasvir, paritaprevir, ritonavir and dasabuvir+ Ribavirin**  **Viekira Pak®+ Copegus®**  **3D+RBV** | **Elbasvir/ Grazoprevir**  **Zepatier®**  **EBR/GZR (± RBV)** |
| **Cost-effective compared to** | **PR** [22] [23] [24] [25] [26]  **PR+BOC** [22] [23]  **PR+TEL** [22] [25] | **PR** [32]  **SOF+RBV***  [35]*****[36] [37]  **SOF/LDV + RBV** [35] | **PR** [23]  **PR+BOC** [23]  **SOF+RBV**  [38] [39] | **PR+ BOC**  [33] [5]  **PR+TEL**  [21] [8] [33] [34]  **SOF+PR**  [5] [4]  **3D (Viekira Pak®)**  [4]  **compared to no treatment**  [40] |  | **PR**  [27] [28] [29]  **PR+BOC**  [27] [29]  **PR+TEL**  [27] [29]  **SOF/LDV**  [41] | **DCV/ASV** [42] | **No treatment** [43]  **SOF/ LDV** [43]  **BOC+PR**  [44] | **PR** [30] [8]  **PR+TEL** [8]  **No treatment** [30] | **DCV/ASV** [6]  **SOF/ LDV**  [7]  **SOF/VEL**  [7]  **PR** [31] |
| **Not Cost-effective compared to** | **SOF+SMV**  [38] [39]  **SOF+DCV**  **(*+RBV)** [35] *****[36] [37] |  |  | **3D** [43]  **EBR/GZR+ RBV**  [7]  **DCV/ASV**  [41]  **SOF+DCV (+RBV)**[35] | **EBR/GZR (± RBV)** [7] | **EBR/GZR** [6]  **OBV/PTV/r** [42] |  | **SOF/ LDV** [4] |  |  |

Table 1: Overview of cost-effectiveness studies classified into interferon and interferon-free treatment regimens; Compared criteria: incremental cost-effectiveness ratio (ICER), treatment cost, drug costs, SVR, gained life-years and QALYs, adverse events, duration, cost-effective ratios (CER)

**References**

1. Bock, J.A., et al., *Cost-effectiveness of IL28Beta genotype-guided protease inhibitor triple therapy versus standard of care treatment in patients with hepatitis C genotypes 2 or 3 infection.* Public Health Genomics, 2014. **17**(5-6): p. 306-19.

2. Brogan, A.J., et al., *Cost-effectiveness of Telaprevir combination therapy for chronic hepatitis C.* PLoS One, 2014. **9**(3): p. e90295.

3. Liu, S., et al., *Sofosbuvir-based treatment regimens for chronic, genotype 1 hepatitis C virus infection in U.S. incarcerated populations: a cost-effectiveness analysis.* Ann Intern Med, 2014. **161**(8): p. 546-53.

4. Chen, G.F., et al., *Will Sofosbuvir/Ledipasvir (Harvoni) Be Cost-Effective and Affordable for Chinese Patients Infected with Hepatitis C Virus? An Economic Analysis Using Real-World Data.* PLoS One, 2016. **11**(6): p. e0155934.

5. Younossi, Z.M., et al., *Cost-effectiveness of all-oral ledipasvir/sofosbuvir regimens in patients with chronic hepatitis C virus genotype 1 infection.* Aliment Pharmacol Ther, 2015. **41**(6): p. 544-63.

6. Chen, P., A. Ma, and Q. Liu, *Cost-Effectiveness of Elbasvir/Grazoprevir Versus Daclatasvir Plus Asunaprevir in Patients with Chronic Hepatitis C Virus Genotype 1b Infection in China.* Clin Drug Investig, 2018. **38**(11): p. 1031-1039.

7. Corman, S., et al., *Cost-Utility of Elbasvir/Grazoprevir in Patients with Chronic Hepatitis C Genotype 1 Infection.* Value Health, 2017. **20**(8): p. 1110-1120.

8. Zhang, S., N.D. Bastian, and P.M. Griffin, *Cost-effectiveness of sofosbuvir-based treatments for chronic hepatitis C in the US.* BMC Gastroenterol, 2015. **15**: p. 98.

9. Elbasha, E.H., et al., *Cost-effectiveness analysis of boceprevir for the treatment of chronic hepatitis C virus genotype 1 infection in Portugal.* Appl Health Econ Health Policy, 2013. **11**(1): p. 65-78.

10. Ferrante, S.A., et al., *Boceprevir for previously untreated patients with chronic hepatitis C Genotype 1 infection: a US-based cost-effectiveness modeling study.* BMC Infect Dis, 2013. **13**: p. 190.

11. Vellopoulou, A., et al., *Cost utility of telaprevir-PR (peginterferon-ribavirin) versus boceprevir-PR and versus PR alone in chronic hepatitis C in The Netherlands.* Appl Health Econ Health Policy, 2014. **12**(6): p. 647-59.

12. Akpo, E.I., K. Cerri, and J. Kleintjens, *Predicting the impact of adverse events and treatment duration on medical resource utilization-related costs in hepatitis C genotype 1 treatment-naive patients receiving antiviral therapy.* Pharmacoeconomics, 2015. **33**(4): p. 409-22.

13. Camma, C., et al., *Cost-effectiveness of boceprevir or telaprevir for previously treated patients with genotype 1 chronic hepatitis C.* J Hepatol, 2013. **59**(4): p. 658-66.

14. Cure, S., et al., *Cost-effectiveness of telaprevir in combination with pegylated interferon alpha and ribavirin in previously untreated chronic hepatitis C genotype 1 patients.* J Med Econ, 2014. **17**(1): p. 65-76.

15. Dan, Y.Y., et al., *Cost-effectiveness of boceprevir co-administration versus pegylated interferon-alpha2b and ribavirin only for patients with hepatitis C genotype 1 in Singapore.* Antivir Ther, 2015. **20**(2): p. 209-16.

16. Petta, S., et al., *Personalized cost-effectiveness of boceprevir-based triple therapy for untreated patients with genotype 1 chronic hepatitis C.* Dig Liver Dis, 2014. **46**(10): p. 936-42.

17. Warren, E., A. Wright, and B. Jones, *Cost-effectiveness of telaprevir in patients with genotype 1 hepatitis C in Australia.* Value Health, 2014. **17**(8): p. 792-800.

18. Saab, S., et al., *Cost-effectiveness analysis of sofosbuvir plus peginterferon/ribavirin in the treatment of chronic hepatitis C virus genotype 1 infection.* Aliment Pharmacol Ther, 2014. **40**(6): p. 657-75.

19. Petta, S., et al., *Cost-effectiveness of sofosbuvir-based triple therapy for untreated patients with genotype 1 chronic hepatitis C.* Hepatology, 2014. **59**(5): p. 1692-705.

20. Kuwabara, H., et al., *Cost-effectiveness analysis of simeprevir in combination with peginterferon and ribavirin for treatment-naive chronic hepatitis C genotype 1 patients in Japan.* J Med Econ, 2015. **18**(7): p. 502-11.

21. Stahmeyer, J.T., et al., *Cost-Effectiveness of Treating Hepatitis C with Sofosbuvir/Ledipasvir in Germany.* PLoS One, 2017. **12**(1): p. e0169401.

22. Cure, S., I. Guerra, and G. Dusheiko, *Cost-effectiveness of sofosbuvir for the treatment of chronic hepatitis C-infected patients.* J Viral Hepat, 2015. **22**(11): p. 882-9.

23. Gissel, C., et al., *Cost-effectiveness of Interferon-free therapy for Hepatitis C in Germany--an application of the efficiency frontier approach.* BMC Infect Dis, 2015. **15**: p. 297.

24. Chung, W., et al., *Cost-effectiveness of sofosbuvir plus ribavirin therapy for hepatitis C virus genotype 2 infection in South Korea.* J Gastroenterol Hepatol, 2019. **34**(4): p. 776-783.

25. Igarashi, A., et al., *Cost-utility analysis of sofosbuvir for the treatment of genotype 2 chronic hepatitis C in Japan.* Curr Med Res Opin, 2017. **33**(1): p. 1-10.

26. Wu, B., Z. Wang, and Q. Xie, *Cost-effectiveness of novel regimens for Chinese patients with chronic hepatitis C.* Curr Med Res Opin, 2019. **35**(5): p. 847-857.

27. Vargas, C.L., et al., *Cost Effectiveness of Daclatasvir/Asunaprevir Versus Peginterferon/Ribavirin and Protease Inhibitors for the Treatment of Hepatitis c Genotype 1b Naive Patients in Chile.* PLoS One, 2015. **10**(11): p. e0141660.

28. Lu, Y., et al., *Cost-effectiveness of daclatasvir plus asunaprevir for chronic hepatitis C genotype 1b treatment-naive patients in China.* PLoS One, 2018. **13**(4): p. e0195117.

29. McEwan, P., et al., *The cost-effectiveness of daclatasvir-based regimens for the treatment of hepatitis C virus genotypes 1 and 4 in the UK.* Eur J Gastroenterol Hepatol, 2016. **28**(2): p. 173-80.

30. Saab, S., et al., *Cost-effectiveness of Ombitasvir/Paritaprevir/Ritonavir, Dasabuvir+Ribavirin for US Post-Liver Transplant Recurrent Genotype 1 HCV.* Liver Int, 2016. **36**(4): p. 515-21.

31. Elbasha, E., et al., *Cost-effectiveness of elbasvir/grazoprevir use in treatment-naive and treatment-experienced patients with hepatitis C virus genotype 1 infection and chronic kidney disease in the United States.* J Viral Hepat, 2017. **24**(4): p. 268-279.

32. Moshyk, A., et al., *Cost-effectiveness of daclatasvir plus sofosbuvir-based regimen for treatment of hepatitis C virus genotype 3 infection in Canada.* J Med Econ, 2016. **19**(2): p. 181-92.

33. Chhatwal, J., Q. Chen, and F. Kanwal, *Why We Should Be Willing to Pay for Hepatitis C Treatment.* Clin Gastroenterol Hepatol, 2015. **13**(10): p. 1711-3.

34. Chhatwal, J., et al., *Cost-effectiveness and budget impact of hepatitis C virus treatment with sofosbuvir and ledipasvir in the United States.* Ann Intern Med, 2015. **162**(6): p. 397-406.

35. Elsisi, G.H., A. Aburawash, and E. Waked, *Cost-Effectiveness Analysis of New HCV Treatments in Egyptian Cirrhotic and Non-Cirrhotic Patients: A Societal Perspective.* Value Health Reg Issues, 2017. **13**: p. 7-15.

36. Restelli, U., et al., *Cost-effectiveness analysis of the use of daclatasvir + sofosbuvir + ribavirin (16 weeks and 12 weeks) vs sofosbuvir + ribavirin (16 weeks and 24 weeks) for the treatment of cirrhotic patients affected with hepatitis C virus genotype 3 in Italy.* Eur J Health Econ, 2018. **19**(1): p. 37-44.

37. Saint-Laurent Thibault, C., et al., *Cost-effectiveness of combination daclatasvir-sofosbuvir for treatment of genotype 3 chronic hepatitis C infection in the United States.* J Med Econ, 2017. **20**(7): p. 692-702.

38. Hagan, L.M., M.S. Sulkowski, and R.F. Schinazi, *Cost analysis of sofosbuvir/ribavirin versus sofosbuvir/simeprevir for genotype 1 hepatitis C virus in interferon-ineligible/intolerant individuals.* Hepatology, 2014. **60**(1): p. 37-45.

39. Pho, M.T. and B.P. Linas, *Valuing cure: bridging cost-effectiveness and coverage decisions for hepatitis C therapy.* Hepatology, 2014. **60**(1): p. 12-4.

40. Adeline, N.J.F., C. Geue, and M.R. Hermami, *Cost-effectiveness of treating hepatitis C in Seychelles.* Pan Afr Med J, 2019. **33**: p. 26.

41. Ward, T., et al., *Assessing the Budget Impact and Economic Outcomes of the Introduction of Daclatasvir + Asunaprevir and Sofosbuvir/Ledipasvir for the Treatment of Chronic Hepatitis C Virus Infection in Japan.* Value Health Reg Issues, 2017. **12**: p. 1-6.

42. Virabhak, S., et al., *Cost-effectiveness of direct-acting antiviral regimen ombitasvir/paritaprevir/ritonavir in treatment-naive and treatment-experienced patients infected with chronic hepatitis C virus genotype 1b in Japan.* J Med Econ, 2016. **19**(12): p. 1144-1156.

43. Chidi, A.P., et al., *Cost-effectiveness of new antiviral regimens for treatment-naive U.S. veterans with hepatitis C.* Hepatology, 2016. **63**(2): p. 428-36.

44. Zhao, Y.J., et al., *Cost-effectiveness of strategy-based approach to treatment of genotype 1 chronic hepatitis C.* J Gastroenterol Hepatol, 2016. **31**(9): p. 1628-37.

**Information on database research**

**1. Search terms used**

- Hepatitis C+ Cost-effectiveness
- Hepatitis C+ cost-effectiveness+ studies
- Hepatitis C cost-effectiveness+ comparison

**2. Database searched**

- Pubmed

**3. Inclusion/ exclusion criteria applied**

Exclusion criteria:

- Treatments of hepatitis and HIV
- Liver transplants (liver transplantation)
- Drug use and hepatitis
- Non-invasive methods for assessment and monitoring
- liver fibrosis (liver fibrosis)
- methods for genotyping
- Screening strategies and screening methods of hepatitis C
- Opioid substitution therapies
- Prevention and management of gastrointestinal and liver disease
- DAA groups are not differentiated by drugs (active ingredients)
- differentiation and effectiveness between groups (F0-F6)

**4. Date search was conducted, and any age restrictions applied to the search**

- May 2020

Records identified through database searching

(n = 1635)

Records after filters were applied

(n = 1334)

Records after duplicates removed

(n = 945)

Full-text articles assessed for eligibility

(n = 135)

Records included

(n = 44)

Filters:

Language (no english) (n= 82)

Species human (n= 219)

Duplicates removed

(n= 389)

Records excluded by title or abstract (n = 810)

Full-text articles excluded (n = 91)

- Treatments of hepatitis and HIV
- Liver transplants (liver transplantation)
- Drug use and hepatitis
- Non-invasive methods for assessment and monitoring
- liver fibrosis (liver fibrosis)
- methods for genotyping
- Screening strategies and screening methods of hepatitis C
- Opioid substitution therapies
- Prevention and management of gastrointestinal and liver disease
- DAA groups are not differentiated by drugs (active ingredients)
- differentiation and effectiveness between groups (F0-F6)

Figure 1: Flow chart of database search
